# Anticoagulation Reversal and Treatment Strategies in Major Bleeding: Update 2016

**DOI:** 10.5811/westjem.2016.3.29294

**Published:** 2016-05-05

**Authors:** Steve Christos, Robin Naples

**Affiliations:** * Presence Resurrection Medical Center, Department of Emergency Medicine, Chicago, Illinois; † Lewis Katz School of Medicine at Temple University, Department of Emergency Medicine, Philadelphia, Pennsylvania

## INTRODUCTION

Anticoagulation is the mainstay of medical treatment, prevention and reduction of recurrent venous thromboembolism, stroke prevention in patients with non-valvular atrial fibrillation, and it reduces the incidence of recurrent ischemic events and death in patients with acute coronary syndrome. Options for anticoagulation have been steadily increasing. Physicians need to be aware of the clinical profile of anticoagulation agents, reversal agents and treatment strategies in the face of major bleeding.

## ANTICOAGULATION TREATMENT OPTIONS

Physicians have numerous anticoagulation treatment options in their arsenal including the traditional injectable indirect factor Xa and IIa (thrombin) inhibitors, which are mediated through an antithrombin dependent mechanism. Examples of injectable indirect factor Xa and IIa inhibitors include unfractionated heparin (UFH), which inhibits factors Xa and to a lesser extent IIa. UFH also inhibits factors XIIa, XIa and IXa. Enoxaparin (Lovenox), a low-molecular-weight-heparin (LMWH), inhibits factors Xa and to a lesser extent IIa. Finally, Fondaparinux (Arixtra) is an exclusive indirect factor Xa inhibitor. Once an injectable indirect factor Xa and IIa inhibitor has been selected, an oral vitamin K antagonist (VKA), traditionally warfarin (Coumadin), is started on the same day. VKAs reduce the synthesis of factors II, VII, IX and X thereby reducing ability to generate active thrombin, rather than inhibition of activated enzymes.

Target-specific oral anticoagulants (TSOACs) or direct oral anticoagulants (DOACs) were developed to provide more stable pharmacokinetic and pharmacodynamic options for oral anticoagulation. Examples of DOACs include the following: direct thrombin inhibitors (DTIs) dabigatran (Pradaxa) and direct factor Xa inhibitors (“Xabans”) rivaroxaban (Xarelto), apixaban (Eliquis) and edoxaban (Lixiana, Savaysa). The DOACs act directly upon Factors IIa (DTIs) or Xa (“Xabans”) without using antithrombin as a mediator.^1^,^2^

Note that each direct factor Xa inhibitor has the letters “Xa” in its spelling.

**Figure 1:**
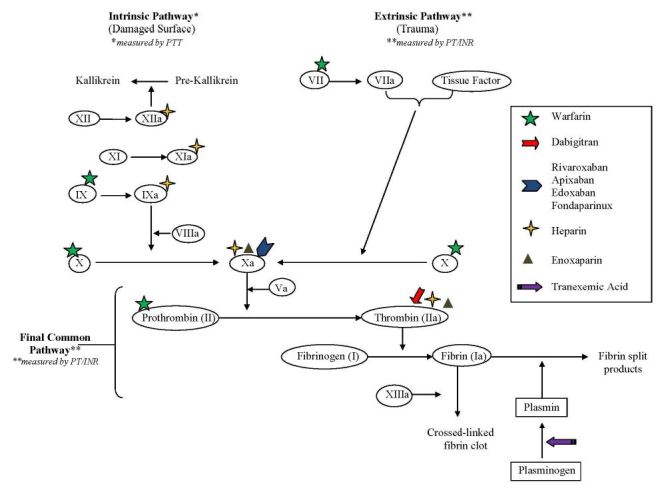
Coagulation cascade and site of action of anticoagulants.

See Figure 1 for an overview of the coagulation cascade and site of action of the anticoagulants.

## CLINICALLY SIGNIFICANT ACUTE HEMORRHAGE

The risk of major bleeding with DOACs is low; however, major life-threatening bleeding can occur. Examples of clinically significant major life-threatening bleeding include intracranial, intraspinal, intraocular, retroperitoneal, intraarticular, pericardial, intramuscular with compartment syndrome or a fall in hemoglobin >2g/dL leading to transfusion. Physicians need to have aggressive and comprehensive anticoagulation reversal and treatment strategies in the face of major bleeding.

## MANAGEMENT OF BLEEDING

1. Discontinue anticoagulant (know half-life) - DOACs have short half-lives (range from 5 to 17 hours), which suggests reversal drugs may not be needed in non-urgent situations; however, in emergency situations such as life-threatening major bleeding or non-elective major surgery anticoagulation reversal strategies should be established.
2. Control active bleeding.
3. Maintain adequate fluid, oxygen and hemodynamic support.
4. Transfuse packed red blood or initiate massive transfusion protocols, if necessary.
5. Consider platelets in patients who are thrombocytopenic or on anti-platelet therapy (aspirin (ASA), clopidogrel (Plavix), prasugrel (Effient), dipyridamole (Persantine) or ticlopidine (Ticlid)
6. Order routine lab tests: complete blood count, basic metabolic panel, liver function tests and disseminated intravascular coagulation panel.
7. Order coagulation testing. (Utility of testing based on anticoagulant is discussed below.)

## COAGULATION ASSAYS

### Activated Partial Thromboplastin Time (aPTT)

Activated partial thromboplastin time (aPTT) is a measure of the intrinsic pathway. Traditionally it has been useful to determine the anticoagulation status of patients receiving UFH. In patients who are taking DOACs, effects on the aPTT are variable. Prolongation of aPTT occurs in a curvilinear fashion in patients taking both IIa and Xa inhibitors; however, the degree of prolongation is dependent upon the reagent used.^3^,^4^,^5^

At therapeutic levels of dabigatran, the clinician should expect the aPTT to be prolonged. Recognize that there may still be clinical anticoagulation effects of dabigatran with a normal aPTT; however, the patient’s serum levels would fall below the therapeutic range (<80μg/L).^4^,^5^

At therapeutic levels of the direct oral “Xabans,” a PTT will not reliably be prolonged. The test is only useful in patients on Xa inhibitors at supra-therapeutic levels.

### Prothrombin Time (PT) and International Normalized Ratio (INR)

Prothrombin time (PT) / international normalized ration (INR) is a measure of the extrinsic and common pathway; it is useful to determine the anticoagulation status of patients receiving VKAs. Similar to aPTT, PT/INR is variably affected by the DOACs. The degree of elevation is specific to the reagent as well as the calibration of the laboratory equipment.^3^–^5^

At therapeutic levels of dabigatran, an abnormality of the PT/INR is not expected. In a patient taking dabigatran, an elevated INR is an indication of serum levels three to four times the upper limit of normal therapeutic concentrations.^4^

Of the direct oral Xa inhibitors, rivaroxaban has the strongest effect on the PT/INR. At therapeutic serum concentrations, one would expect both rivaroxaban and edoxaban to cause elevation of the PT/INR.^3^,^5^,^6^ Apixaban weakly affects the PT/INR levels.^5^ Similar to stated above, a normal PT/INR does not exclude some degree of anticoagulant effect, but merely indicates a level below that expected at therapeutic dosing.

### Thrombin Time (TT); Also Known as Thrombin Clotting Time

Thrombin time (TT) directly assesses the activity of thrombin. This test is useful in patients receiving dabigatran (Pradaxa). A normal TT excludes dabigatran activity; the degree of elevation of the TT is not a direct correlate of serum levels*.*^4^

### Chromogenic Anti-Factor Xa

Chromogenic anti-factor Xa testing measures the concentration of anticoagulants that inhibit factor Xa. This test is useful in patients receiving LMWHs, fondaparinux (Arixtra) and direct oral factor “Xabans”.

## ANTICOAGULATION REVERSAL AND TREATMENT OPTIONS IN MAJOR BLEEDING

**Figure 2:**
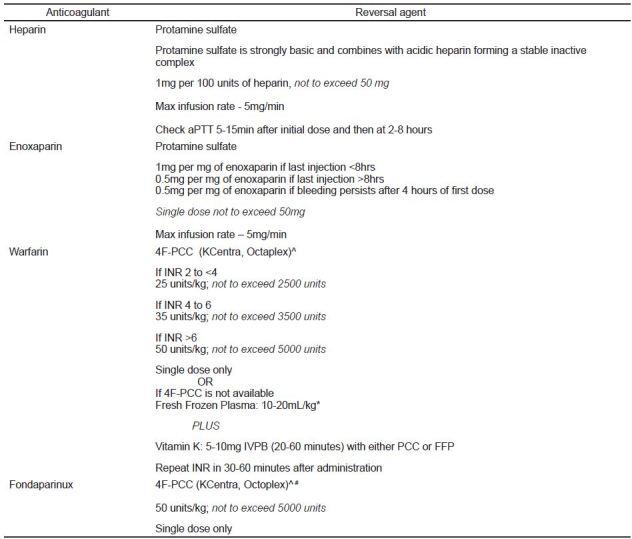
Reversal strategies for conventional anticoagulants in patients with significant bleeding. *PCC*, prothrombin complex concentrates; *INR*, international normalized ratio; IVPB, intravenous piggyback; *FFP*, fresh frozen plasma ^4F-PCC contains heparin and is contraindicated in patients with a history of heparin induced thrombocytopenia. ^*^4F-PCC has factor concentrations 25 × greater than FFP. Reversal of INR with 4F-PCC is within 30–60 minutes versus 6–24 hours in FFP. Risk of volume overload and transfusion-related acute lung injury (TRALI) with FFP. Thrombotic event rate 8.7% for 4F-PCC vs. 5.5% with FFP. ^#^Off-label use.

Anticoagulation reversal and treatment options in major bleeding include protamine, phytonadione (Vitamin K), hemodialysis, oral-activated charcoal, antifibrinolytic agents including tranexamic acid, desmopressin, blood products including packed red blood cells (PRBCs) and platelets, prothrombin complex concentrates (PCCs), and specific reversal agents. See Figure 2 for reversal strategies of conventional anticoagulants in patients with significant bleeding.

**Figure 3:**
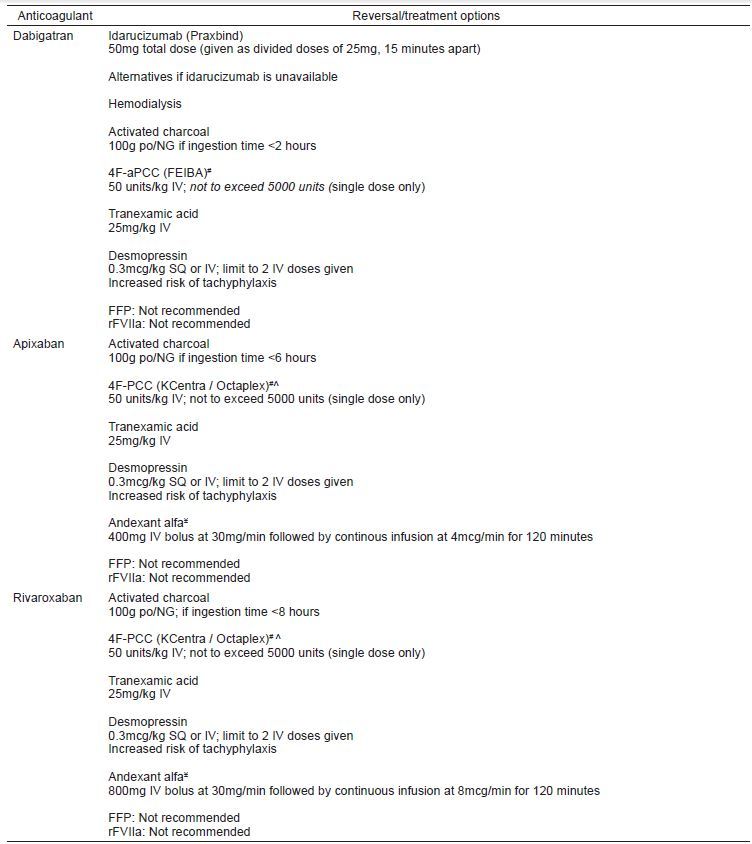
Reversal of direct oral anticoagulants (DOACs) in patients with significant bleeding. *FFP*, fresh frozen plasma; *rFVIIa*, Recombinant human Factor VIIa; *PCC*, prothrombin complex concentrates; *FEIBA*, Factor Eight Inhibitor Bypassing Activity; *NG*, nasogastric #Off label use. ^4F-PCC contains heparin and is contraindicated in patients with a history of heparin induced thrombocytopenia. ¥Not currently available on market. FDA trials ongoing. Dosing based on published Phase 3 trial.

Given most clinicians’ apprehension and relative inexperience with reversal of the DOACs, below we will discuss in detail the risks and benefits of different treatment options. (See summary - reversal of direct oral anticoagulants in patients with significant bleeding, Figure 3,)^7^–^16^

## SPECIFIC REVERSAL AGENTS

### Idarucizumab (Praxbind)

**Figure 4:**
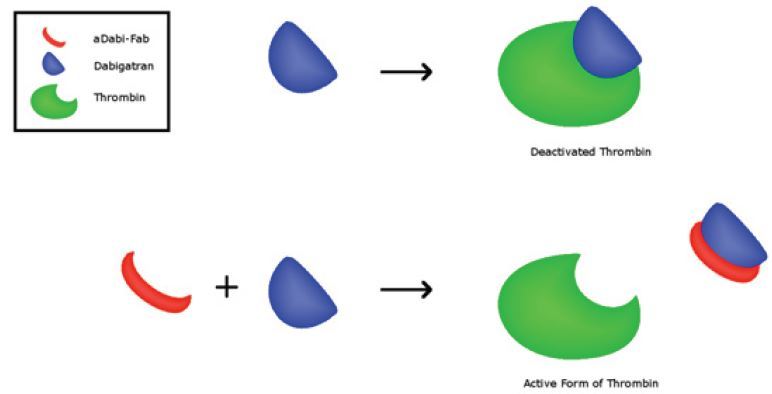
Fab fragments (aDabi-Fab) reversal effects on dabigatran.

At the current time, idarucizumab is the only FDA-approved agent for reversal agent of the direct oral anticoagulants, and it only works on dabigatran. It is a monoclonal antibody fragment that binds with high affinity dabigatran (Figure 4).^17^ It has been shown in both *in vitro* studies in health volunteers to rapidly reverse the coagulation effects of dabigatran.^7^,^8^ In the RE-VERSE AD clinical trial in patients with active bleeding, idarucizumab was shown to reverse the anticoagulant effect in laboratory testing, an effect that was evident within minutes.^8^ Hemostasis was reported as being improved without significant thrombosis risk; however the improvement in patient outcomes (20% mortality in the group) is unclear.[8] If available, idarucizumab is recommended for reversal of anticoagulant effects for patients taking dabigatran.

### Andexanet Alfa

**Figure 5:**
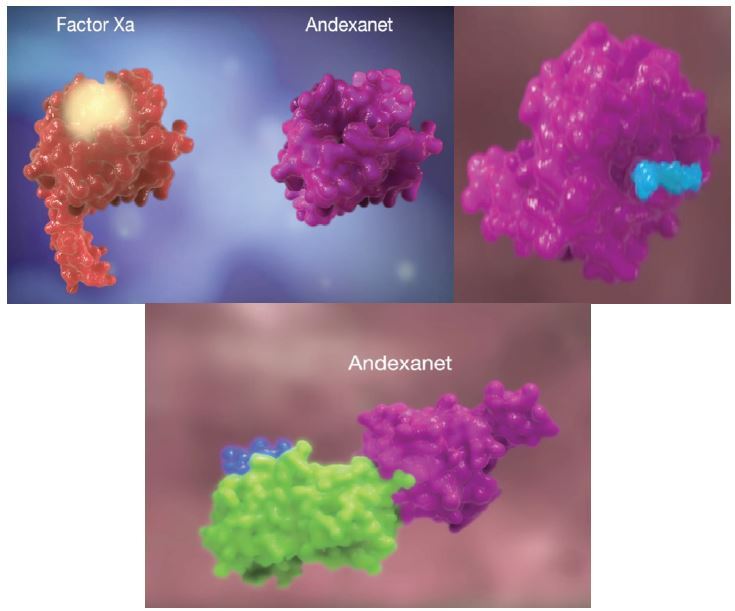
Andexanet alfa mechanism of action video.

Andexanet alfa acts as a decoy to target and sequester with high specificity both oral and injectable factor Xa inhibitors (Figure 5).^18^ It has been developed as an antidote to reverse the anticoagulant activity of oral direct (apixaban, edoxaban, and rivaroxaban) and injectable indirect (enoxaparin and fondaparinux) factor “Xabans.” Currently its manufacturer is pursuing FDA approval and it is in phase 4. ANNEXA-A and ANNEXA-R were trials published from phase 3 data that showed rapid reversal of anticoagulation effects in healthy volunteers given therapeutic doses of apixaban and rivaroxaban.^9^ This drug is not currently available.

### Aripazine

Aripazine is a small, synthetic molecule with broad reversal activity administered as a single intravenous bolus dose. It binds to oral factor Xa inhibitors and DTIs, as well as UFH and LMWH via non-covalent bonding and charge-charge interactions to neutralize anticoagulation and bleeding.^11^ This drug is not currently available.

### Prothrombin Complex Concentrates (PCCs)

Prothrombin complex concentrates have been developed to contain highly concentrated coagulation factors along with antithrombotic agents. Depending on the agent used, they may contain three factors (II, IX, X) or four factors (II, VII, IX, X). Four-factor (4F-) PCC may be inactive (4F-PCC; K-centra, Octaplex) or active (4F-aPCC; FEIBA); this is determined by the Factor VII component.

PCCs have been shown to be successful in the reversal of VKAs and are considered first line treatment in patients with major bleeding on VKAs.^11^ Clotting factors are 25 times more concentrated than fresh frozen plasma (FFP) and INR reverses within minutes (15–20 minutes), while it may take FFP 6 to 24 hours. PCCs are available by IV immediately versus delay needed with FFP, and the risk of volume overload and transfusion-related acute lung injury (TRALI) with FFP is not seen with PCCs. Disadvantages of PCCs vs FFP include a small but real pro-thrombotic risk, availability at some institutions and cost. The cost for a course of treatment with FFP in a patient with warfarin-associated intracranial hemorrhage (ICH) and an INR of 3.0 has been estimated to be between $200 and $400 (U.S. dollars) vs $1,000 to $2,000 (U.S. dollars) for a course of treatment with PCC.^19^

*In vitro* human studies of healthy volunteers who have received oral direct IIa and Xa inhibitors have shown normalization of coagulation testing after receiving both 4F-PCC and 4F-aPCC.^11^,^12^ A variety of animal studies have been performed with dabigatran, rivaroxaban and apixaban.^12^–^16^ These studies have yielded variable results in improvement of bleeding times and overall blood loss. There have been no studies evaluating the effectiveness of four-factor PCCs in patients with active bleeding on DOACs. Given the available in vitro and animal literature, 4F-PCC (K-Centra, Octaplex) is probably beneficial in patients taking oral direct “Xabans” with less benefit in patients on an oral direct IIa inhibitor (dabigatran). The contrary is true of 4F-aPCC (FEIBA); 4F-aPCC is probably beneficial in patients on dabigatran, with less benefit in those on the “Xabans.” With use of both four-factor PCCs, the risk of thrombosis needs to be considered against the benefit of hemorrhage control. Studies of 3F-PCC have not been performed in either *in vitro* or in animal models and thus, no recommendation can be made regarding its use in bleeding patient on DOACs.

### Recombinant Human Factor VIIa (rFVIIa, NovoSeven)

Recombinant human Factor VIIa (rFVIIa) activates the coagulation cascade via the extrinsic pathway and has been used off label in the reversal of VKAs. Similar to PCCs, *in vitro* human and bleeding animal model studies have been performed to evaluated the benefit of this therapy in patients on DOACs.^10^,^16^ While the above-mentioned studies have shown improvement in laboratory testing, bleeding times and blood loss, the dose required to achieve benefit far exceeds the normal dosing of rFVIIa. Because of the high doses required and the concern for the possibility of thrombotic sequelae, rFVIIa is not recommended for the treatment of bleeding in patients on TSOAC therapy.

### Fresh Frozen Plasma (FFP)

Fresh frozen plasma (FFP) is derived from whole blood and thus, contains all inactive components of the coagulation cascade in physiologic concentrations. FFP has traditionally been considered an option in patients on oral anticoagulants (*e.g.* VKAs) Unlike VKAs, the DOACs do not inhibit overall production of inactive coagulation components, but only bind to specific active factors. Therefore, treatment of significant bleeding in a patient taking DOACs is not aimed at repleting diminished factors. The relative concentration of coagulation factors in FFP is diminutive compared to the PCC products such that one would need over two liters of FFP to approach similar concentrations. The time to thaw and transfuse this volume of FFP also is a disadvantage. For these reasons, FFP is not recommended for reversal of DOACs unless no other agent is available.^10^,^16^ It should continue to be used as dictated by your institution’s massive transfusion protocol.

### Tranexamic Acid (TXA)

Tranexamic acid inhibits fibrinolysis by inhibiting the binding of plasma to fibrin. While no studies have looked at the efficacy of TXA, the cost and overall risk of administering therapy (adverse reaction, thrombotic sequelae) is low. Therefore, the recommendation is that TXA should be considered for reversal of bleeding in patients taking DOACs.

### Desmopressin (DDAVP)

Desmopressin is a synthetic analogue of vasopressin. It affects thrombosis by stimulating the release of von Willebrand factor (vWF) and increasing production of factor VIII. Similar to TXA, the overall cost and risk of administration of desmopressin is low. Therefore, it should be considered for use in the treatment of significant bleeding in patients on DOACs.

### Hemodialysis

Dabigatran excretion is 80–85% renal which makes hemodialysis an option.

Direct factor Xa oral inhibitors are mainly protein bound with only 25 to 35% renal excretion, therefore hemodialysis is not an option for the direct factor “Xabans”.^10^

### Oral Activated Charcoal

Oral activated charcoal, 100gm PO/NG ×1, is an option to reduce absorption for all DOACs in the appropriate patient. Charcoal can be considered if the dose was taken within eight hours for rivaroxaban, six hours apixaban and within two hours of ingestion for edoxaban and dabigatran.

## CONCLUSION

Physicians need to effectively manage major bleeding in patients on anticoagulants. Understanding the clinical profiles of anticoagulants and developing treatment and reversal strategies will help physicians effectively manage this life-threatening emergency. While physicians are comfortable with the use of vitamin K, fresh frozen plasma and protamine, the use of prothrombin complex concentrates and future antidotes hold promise in improving the outcomes of patients who require emergent reversal of their anticoagulant therapy.
